# Spiritually-oriented therapy for endogenous mental patients with comorbid addictive disorders

**DOI:** 10.1192/j.eurpsy.2021.2104

**Published:** 2021-08-13

**Authors:** V. Mitikhin, A. Magay

**Affiliations:** Department Of Mental Health Support Systems Research Centre, Mental Health Research Centre, Moscow, Russian Federation

**Keywords:** schizophrénia, comorbid, addictive, effectiveness

## Abstract

**Introduction:**

Spirituality and religious commitment have a “protector” function among mental health patients who abuse psychoactive substances. The main task of spiritually-oriented therapy is not only to reactivate the internal control of a person, but to actualize their experience of communication with God before everything else.

**Objectives:**

Studying the influence of spiritual life-related factors on efficiency of therapy of psychiatric co-morbidities.

**Methods:**

Clinical and psychopathological, clinical follow-up, pathopsychological and statistic.

**Results:**

The research covered 26 patients (the main group) diagnosed with paroxysmal schizophrenia and schizo-affective psychosis in the prospective follow-up with alcohol addiction. All patients practiced Orthodox worldviews though to a different extent, and have been participating in the spiritually-oriented rehabilitation with a family-oriented module for two years. During psychosocial rehabilitation the patients took group and individual training with a multidisciplinary team of experts: psychiatrists, clinical psychologists, specialists in sociotherapy and members of the clergy. The rehabilitation employed the principles of therapeutic community, systematic family approach [Zoricic Z., 2019], notions of coping behavior or coping strategies [Verhagen P., 2019, Pargament, K.I. et al, 2014] as well as spiritually-oriented models of assistance to patients (for example, the religion-oriented strategy of forgiveness based on REACH model [Worthington E. L. et al, 2016]).

**Conclusions:**

Development of a lengthy remission is dependent on changing lifestyle and patterns, and spiritual labor of penance and forgiveness is just as important.

**Disclosure:**

No significant relationships.

